# Nitroxidergic modulation of behavioural, cardiovascular and immune responses, and brain NADPH diaphorase activity upon morphine tolerance/dependence in rats

**DOI:** 10.1080/13102818.2014.990924

**Published:** 2014-12-13

**Authors:** Ana Tsakova, Slavina Surcheva, Katerina Simeonova, Iskra Altankova, Tsvetanka Marinova, Kamen Usunoff, Mila Vlaskovska

**Affiliations:** ^a^ Department of Pharmacology and Toxicology, Medical Faculty, Medical University of Sofia, Sofia, Bulgaria; ^b^ Department of Biology, Medical Genetics and Microbiology, Faculty of Medicine, Sofia University “St. Kliment Ohridski”, Sofia, Bulgaria; ^c^ Department of Anatomy, Histology and Embryology, Medical Faculty, Medical University of Sofia, Sofia, Bulgaria

**Keywords:** morphine tolerance/dependence, L-NAME, behaviour, cardiovascular, thymocyte apoptosis, lymphocyte proliferation, NOS

## Abstract

Opioid and non-opioid effects of acute and chronic morphine administration on behaviour, cardiovascular responses, cell proliferation and apoptosis and nitric-oxide synthase (NOS) activity were studied in rats. A novel score-point scale was introduced to quantify the signs of opioid withdrawal syndrome. NOS inhibitor L-NAME (N^G^-nitro-L-arginine methyl ester) was applied to reveal the role of NOS/NO pathway in the modulation of morphine-induced *in vivo* and *in vitro* responses. The obtained data showed that chronic co-administration of L-NAME drastically attenuated naloxone-precipitated withdrawal syndrome and prevented the development of morphine tolerance to cardiovascular action of morphine. The apoptotic process was very much restricted by L-NAME supplementation of chronic morphine treatment, which resulted in few apoptotic cells, less low molecular weight genomic DNA and preservation of high molecular weight non-fragmented genomic DNA. The study provides new data for nitroxidergic modulation of opioid tolerance and dependence.

## Introduction

Opioids are widely used for treatment of severe pain. The acute opioid action in opioid-naive individuals is manifested in analgesic and/or euphoric effects. Long-term opioid administration may result in tolerance and dependence is mostly manifested in compulsory drug-seeking behaviour and/or opioid withdrawal syndrome. Opioid dependence/withdrawal is a complex mono-etiological, multi-pathogenesis stress reaction of the organism at cellular and integrative levels. The central nervous system (CNS) is the primary target of opioid action and the mesolimbic system is the main component of the brain reward system.[1] Ventrolateral periaqueductal grey matter (PAG) is the principal relay station of pain modulation [2] and PAG neurons constitute neuronal circuits that are integrated in the descending pain modulation pathway.

It is well known that nitroxidergic mechanisms could modulate opioid-induced reactions and vice versa, opioids could change nitric-oxide synthase (NOS) activity and, thereby, the synthesis and release of nitric oxide (NO). Most authors agree that NOS/NO are involved in development of tolerance to morphine analgesic action [3] and manifestation of opioid abstinence syndrome.[4] NO could diminish the morphine influx into the brain and spinal cord structures that are specialized in the processing of nociceptive stimuli.[5] Attenuation,[6] augmentation [7] or no change [8] in morphine-induced analgesia, acceleration of morphine tolerance [9] or suppression of morphine uptake into specific brain zones [5] are all found after acute and chronic L-arginine administration. There is ample evidence that NOS inhibitors can prevent the development of tolerance to morphine [10] and attenuate the dependence/withdrawal syndrome.[11] The data reported for effects of various NOS inhibitors on nociception vary from potentiation,[12] to no effect [13] or significant attenuation.[7] Such contradictory and fragmentary data make it difficult to draw a consistent scheme about the influence of NOS/NO on opioid-evoked reactions. On the other hand, there is consistent evidence that acute morphine administration could increase NOS activity in CNS.[14] NOS activity is potentiated during opioid withdrawal [15] but remains unchanged upon opioid tolerance.[16] Despite the evidence for massive NOS-positive staining in midbrain nuclei, further studies are needed to verify the opioid effects on brain NOS activity.

It is a common clinical knowledge that therapeutic doses of opioids produce mild hypotension and bradycardia in naive supine subjects primarily due to histamine release from mast cells and blotting of reflex vasoconstriction caused by increased partial pressure of CO_2_ and that orthostatic fainting may occur in head-up posture.[17] Intravenous administration of morphine causes centrally mediated increase in vagal outflow to the heart leading to atropine-sensitive bradycardia, right ventricular preload, ino- and chronotropy. It has been suggested that most cardiovascular effects of opioids are mediated by opioid receptor independent mechanisms.[18] The mechanisms of opioid action in the cardiovascular system have not so far been explicitly characterized at cellular and integrative level.

A trivial explanation of opioid action in the immune system is mild immunosuppression and increased pro-inflammatory and metastatic potential. There are also data that opioids could reverse the pain-induced immunosuppression and can increase the tumour metastatic potential.[19] Opioid withdrawal has also been reported to aggravate the immune dysfunction in immunocompromised patients who sustain severe pain, as revealed clinically by higher susceptibility to HIV or tuberculosis infection. The central acute opioid action in the immune system is mediated by activation of sympathetic nervous system, whereas the central chronic opioid suppression of the immune system involves the hypothalamus–pituitary–adrenal axis [20] by μ-opioid-receptors-mediated NOS/NO-dependent inhibition of NF-κB.[21] Thus, the overall picture of the reported opioid immune effects is still rather fragmentary. Further study of opioid action in the immune system would help rational and individualized therapy of patients with cancer or higher risk of infection.

There is evidence suggesting that opioids could evoke cell proliferation and cell death via pathways other than adenylyl cyclase–cAMP–protein kinase A. It has been shown that morphine treatment during embryogenesis can prevent apoptosis [22] and can stimulate cell proliferation [23] associated with increased external stimulus-activated protein kinase expression and/or phosphorylation.[24] Opioid tolerance could induce apoptosis of GABAergic inhibitory neurons, thus compromising the endogenic mechanisms of pain inhibition.[25] Naloxone has been shown to prevent apoptosis of neurons but fails to prevent apoptosis of malignant cells. This finding might have useful clinical implications. Chronic treatment with high dose morphine plus low dose naloxone might be a therapeutic option that would combine favourable tumour suppression with reduced neuronal toxicity in cancer patients.[25]

The aim of the present research was to study the nitroxidergic modulation of different analgesic and non-analgesic effects induced by acute and chronic administration of morphine in *in vivo* and *in vitro* models in rats. The focus of study was on *in vivo* morphine tolerance, dependence/withdrawal revealed by several behavioural and cardiovascular symptoms, *in vitro* lymphocyte proliferation and thymocyte apoptosis/DNA fragmentation before and after NOS inhibition, as well as the nicotinamide adenine dinucleotide phosphate (NADPH) diaphorase activity in brain nuclei that participate in pain processing.

## Animals and methods

### Animals

Male Wistar rats (220 –240 g, body weight) were obtained from the animal house of the Medical University of Sofia and were housed in a controlled environment at a natural day/night cycle at 22 ± 2 °C and *at libitum* access to water and chock food. By the end of the *in vivo* experiments rats were sacrificed with an overdose of CO_2_ gas and exsanquination or by air embolism. The substances (Sigma–Aldrich, Germany) were administered *in vivo* as saline solutions in a volume of 1 mL/kg, or 3 mL/kg (L-arginine). The research protocol and manipulations were approved by the Ethics Committee of the Medical University of Sofia.

### Behaviour

In a series of experiments, the tolerance to the analgesic action of morphine and morphine ED_50_ were studied in unrestrained rats grouped (G) at random (8–10 rats per G) for acute morphine (5 mg/kg, s.c.) or morphine (5 mg/kg, s.c.) plus L-NAME (N^G^-nitro-L-arginine methyl ester) (15 mg/kg, i.p.) administration. In another series of experiments rats were distributed at random (8–10 rats per G) in groups of long-term drug treatment: (group 1/G1) morphine injected s.c. twice at a daily dose of 5 mg/kg that increased every other day by 5 mg/kg up to 30 mg/kg at 9:00 a.m. and 5:00 p.m. for 11 consecutive days; (G2) L-NAME injected i.p. at a daily dose of 5 mg/kg at 8:30 a.m. for 11 consecutive days; (G3) morphine injected as in G1 plus L-NAME injected as in G2; (G4) saline injected at a daily dose of 1.0 mL/kg as in G1; (G5) morphine injected as in G1 plus naloxone injected i.p. at a single dose of 5 mg/kg at 10:00 a.m. on day 11, (G6) morphine plus L-NAME injected as in G3 plus naloxone injected as in G5; (G7) morphine injected s.c. at a single dose of 20 mg/kg at 9:00 a.m.; (G8) naloxone injected i.p. at a single dose of 5 mg/kg at 10:00 a.m.

The nociception threshold was determined using radiant heath tail-flick test.[26] The response latency to radiant heath stimulus (tail withdrawal reflex) was measured by tail-flick analgesimeter (Ugo Basile, Italy) at 20 s preset cut-off time. A novel cumulative score-point scale upgrading previous studies [4,11] was developed for quantification of symptoms of post-treatment or naloxone-precipitated opioid withdrawal behaviour (Table 1). The symptoms of opioid dependence/withdrawal of rats unrestrained under large transparent glass funnels were scaled by two witnesses who were unaware of the preceding drug treatment through three consecutive 10 min observation periods. The score point of the second observation period, the cumulative score point of the entire observation time and the mean group score point were determined. The score point was limited to an appropriate upper-range maximum. The behaviour observation was conducted at midday time. After completion of the behavioural experiments, rats were selected at random from each group G1–G4 for *in vitro* studies of lymphocyte proliferation and apoptosis/DNA fragmentation (3 rats/G) and from each group G1–G8 for *in vitro* histochemistry of brain NADPH diaphorase (3 rats/G).

**Table 1.** Score-point scale of opioid dependence/withdrawal symptoms.

Symptoms	Score points	Maximum score points/10 min
Defecation (normally formed stool)	1 for each 4 droppings	2
Salivation, lacrimation	1 for each	2
Back-fur piloerection, ptosis	1 for each leastwise 60 s non-stop	5
Sniffing, grooming, gnawing	1 for each leastwise 60 s non-stop	5
Teeth chattering/mastication/chew	1 for each leastwise 60 s non-stop	5
Penile licking	1 for leastwise 30 s non-stop	5
Vocalization on touch at 2 min	1 for each episode	5
Aggressive behaviour on touch at 2 min	1 for each episode	5
Post-treatment body weight loss	1 for each 2.5% bw loss on next day	8
Escape attempts	1 for each 2 attempts	8
Wet-dog shakes	1 for each leastwise 60 s non-stop	8
Hyperactivity, exploration, rearing	1 for each leastwise 60 s non-stop	8
Writhing/wrench/twist	1 for each	8
Diarrhoea (loosely formed stool)	1 for each episode	8
Wax-figure posture	1 for each leastwise 60 s non-stop	10
Prostration	1 for each leastwise 30 s non-stop episode	10

### Cardiovascular system

Rats were anesthetized (pentobarbital, 40 mg/kg, i.p., Apoteksbolaget, Sweden, plus calipsol, 2.5 mg/kg, i.p., Richter, Hungary). Distal abdominal aorta and right iliac vein were catheterized (PE 10, Portex, USA) for monitoring of arterial blood pressure (BP) and heart rate (HR) and administration of drugs. After 2 day recuperation, rats were at random distributed in groups (D, 5–6 rats per D) for drug administration: (D1) morphine administered at a dose of 0.5 mg/kg 24 h and 72 h after catheterization; (D2) L-NAME administered at a dose of 6 mg/kg 15 min prior to morphine applied as in D1; (D3) L-arginine administered at a dose of 300 mg/kg 15 min prior to morphine applied as in D1; (D4) L-arginine administered as in D3 20 min prior to L-NAME and morphine administered as in D2. BP and HR of unrestrained rats were recorded non-stop over a 72 h period by means of BP transducer (P23ID Statham, USA) connected to an MP 100 WSW system (Biopac Systems, USA). Data were processed by AcqKnowledge III, 3.2 software for continuous monitoring of biological signals (Biopac Systems, USA). Drug effects on BP and HR were monitored in restrained rats (Ballman type KN 326 restrainer III, Natsume, Japan) and determined as per cent change (±) of systolic BP (SBP), diastolic BP (DBP), mean BP (MBP) and HR.

### Concanavalin A-induced lymphocyte proliferation

Lien of rats from each group G1–G4 were collected, minced and disrupted through a steel mesh. The cell suspension was rinsed in sterile phosphate buffer (PB) and centrifuged at 1000 *g* for 10 min at 23 °C. The cell pellet was suspended in 20 mL of sterile PB, distributed into 5 mL histopaque tubes and centrifuged at 400 *g* for 30 min at 23 °C. Interface white cells were harvested, washed twice with sterile PB and resuspended in sterile RPMI (Roswell Park Memorial Institute) medium containing 10% fetal calf serum and penicillin/streptomycin (100 U/mL) to a final density of 2 × 10^6^ cells/mL. To avoid development of “*in vitro* withdrawal” [27], morphine (1 μmol/L) or L-NAME (5 μmol/L) were present throughout to mirror the *in vivo* drug treatment. Aliquots (100 μL) from cell suspensions obtained from rats treated with morphine, L-NAME, morphine plus L-NAME or saline were inoculated in triplicate wells on flat-bottom cell culture plates with addition of concanavalin A at a concentration of 125 or 500 μg/mL. The plates were incubated in 5% CO_2_ humidified atmosphere for 48 h at 37 °C. Each well was loaded with ^3^H-thymidine (1 μC/well) 20 h prior to cell harvest (Cell harvester, Flow Laboratories, UK) on specialized blot discs (Watmann, USA). Individual discs were dissolved in scintillation solution and ^3^H-radioactivity of the samples was measured (Beta Rack LKB, Sweden) and expressed as disintegrations per minute (DPM). Proliferation index was calculated as the ratio DPM test sample (drug)/DPM control sample (saline).

### Thymocyte apoptosis/DNA fragmentation

Thymus gland of rats in each group G1–G4 were collected, freed of connective tissue and partitioned. Thymus fragments were fixed in 2.5% glutaraldehyde in 0.1 mmol/L cacodylate buffer (pH 7.4) for 1 h at 4 °C, post-fixed in 1% OsO_4_ for 2 h and cut. Semi-thin sections (100 μm) were stained with 1% toluidine blue for light microscopy (LM). Ultra-thin sections (50 nm) were stained with 1% uranyl acetate and lead acetate for transmission electron microscopy (EM). Thymus cell suspension was pelleted and resuspended in 5 mL buffer containing 75 mmol/L of NaCl and 25 mmol/L of NaEDTA (pH 8.0) for 30 min, followed by incubation at 35 °C in the presence of protease K (100 mg/mL) for 6 h. DNA in viscous solution was extracted twice with phenol and once with 50% 2-propanol at 20 °C for 3 h. The extract was digested with RNAase (50 mg/mL) at 37 °C for 2 h. LM, EM, indirect anexin V immunofluorescence, terminal deoxynucleotidyl transferase dUTP nick end labeling (TUNEL) and indirect immunoperoxidase staining were applied as described previously.[28] Agarose gel electrophoresis of fragmented DNA in 1% agarose gel in 90 mmol/L TRIS-borate buffer (pH 8.0) stained with ethidium bromide (1 mg/mL) was visualized under ultraviolet light as described earlier.[29] Annexin V-FL (Santa Cruz Biotechnology, USA), Annexin V-FITC (Boehringer, Germany), TUNEL In Situ Cell Death Detection Kit (Boehringer, Germany) were used. Axioskop 20 light microscope (Zeiss Opton, Germany) and Hitachi H500 electron microscope (Hitachi, Japan) were used for viewing.

### Brain NADPH diaphorase

Rats were anesthetized (Aether pro narcosis, Sopharma, Bulgaria) and perfused transcardially with 0.05 mol/L PB for 5 min, followed by 20 min fixative perfusion of 4% paraformaldehyde and 0.15% picric acid in 0.1 mol/L PB. Brain of rats from each group G1–G8 was removed, post-fixed in the same fixative for 24 h, submersed overnight in 20% sucrose in 0.1 mol/L PB and cut into serial coronal slices (30 μm) on freezing microtome (Reichert Young, Austria). Diaphosase histochemistry was performed according to modified Scherer–Singler procedure.[30] Free floating tissue slices containing PAG, dorsal raphe nucleus (DRN), pedunculopontine tegmental nucleus (PPN) and laterodorsal tegmental nucleus (LDT) were rinsed in 0.8% Triton X-100 (Fluka, Germany) in 0.1 mol/L Tris/HCl buffer (pH 7.4) for 30 min and incubated in 10 mL Tris-HCl buffer containing 4 mg of NADPH (Sigma, Germany) and 10 mg of Nitroblue tetrazolium (Fluka, Germany) for 2 h at 37 °C. Slices were washed out in PB and mounted onto chrome-gelatin coated slides, air-dried for 24 h, dehydrated and embedded in Etalan (Merck, Germany). Light photomicroscope (Jenaval, Jena Zeiss, Germany) was used for viewing. Stain intensity was evaluated by computer-assisted image analysis in optical density arbitrary units (OD) in the range from 0 (absolute transparency) to 254 (absolute opacity) OD. Diaphorase positive cells were absent in all slices incubated identically in NADPH-free medium.

### Statistical analysis

Mean values ± standard error of mean were calculated and the results were evaluated by analysis of variance at *P* ≤ 0.05 using statistical package GraphPad InStat (GraphPad Inc., USA).

## Results and discussion

### Behaviour

It was found that morphine ED_50_ in naive rats was 4.9 ± 0.6 mg/kg which increased to 17.2 ± 1.6 mg/kg in rats treated with morphine plus L-NAME. The quantitative analysis of behaviour revealed no differences between group G4 and groups G2 and G8. The aggregate score points were 12.0 ± 2.6 in saline treated rats (group G4) compared to 17.0 ± 3.7 and 13.5 ± 1.9 in rats treated with L-NAME (group G2) and naloxone (group G8), respectively (Table 2). Acute morphine treatment (group G1, day 1) evoked typical behavioural effects: stereotypic motor activity and Straub tail reflex alternating with catalepsy. Pretreatment with L-NAME (group G3, day 1) did not change the behavioural effects of acute morphine and morphine-induced analgesia. The control latency was 5.62 s ± 0.56 s (group G4) which increased to 14.8 ± 1.3 s and 15.3 ± 1.0 s by acute morphine treatment or acute morphine plus L-NAME pretreatment, respectively. After five days of chronic morphine treatment (group G1), tolerance to the analgesic action of morphine occurred and latency decreased to 7.45 ± 1.01 s. Co-administration of L-NAME (group G3) prevented the development of morphine tolerance, which was revealed by the increase in latency up to 13.8 ± 1.24 s. The response latency to radiant heat was very similar over the following five days of morphine treatment, accompanying progressively aggravated symptoms of drug dependence prior to subsequent morphine administration. Main abstinence symptoms of vocalization upon touch, irritability/aggression and grooming were more evident in group G1 than in group G3. The behavioural difference between group G1 and group G3 was most prominently demonstrated during naloxone-precipitated withdrawal. The withdrawal syndrome in group G5 was dominated by writhing, teeth chattering, ptosis, back-fur piloerection, vocalization upon touch, wax-figure posture, escape attempts, diarrhoea and severe prostration. This contrasted with much alleviated prostration, diarrhoea, wax-figure posture and vocalization upon touch found in group G6. In this group, withdrawal syndrome was dominated by sniffing, hyperactivity (rearing) and writhing. After seven days of chronic morphine administration, the body weight loss for rats in group G5 was 10.1 ± 1.1 g, which diminished to 7.4 ± 0.9 g for rats in group G6. The analysis revealed that the aggregate score-point value for abstinent rats in group G1 was 42.5 ± 6.8, and 12.0 ± 2.6 for rats in group G4. Naloxone-precipitated withdrawal tripled the aggregate score-point value for morphine abstinent rats in group G5 to 126.0 ± 19.3. However, the aggregate score-point value for naloxone-precipitated withdrawal in rats treated with morphine plus L-NAME in group G6 was 39.5 ± 7.4, which was significantly lower than the corresponding aggregate score-point value for the morphine treated rats in group G5. The quantitative evaluation of behaviour is presented in Table 2.

**Table 2.** Quantitative evaluation of behavioural symptoms upon various drug treatments.

Treatment (group)	Points/10 min	Points/30 min
Chronic morphine (G1)	17.5 ± 1.1^a^	42.5 ± 6.8^a^
Chronic L-NAME (G2)	6.0 ± 0.9	17.0 ± 3.7
Chronic morphine plus L-NAME (G3)	6.0 ± 0.9	17.0 ± 4.2
Morphine withdrawal (G5)	47.0 ± 4.3^b^	126.0 ± 19.3^b^
L-NAME plus morphine withdrawal (G6)	14.5V 2.4^c,d^	39.5 ± 7.4^c,d^
Acute morphine (G7)	7.2 ± 1.2	19.0 ± 6.1
Acute naloxone (G8)	4.5 ± 0.4	13.5 ± 1.9

^a^ *P* ≤ 0.01 vs. G4; ^b^ *P* ≤ 0.001 vs. G1; ^c^ *P* ≤ 0.05 vs. G4; ^d^ *P* ≤ 0.001 vs. G5.

The present findings show that pretreatment with L-NAME does not change the characteristic feature and magnitude of behavioural symptoms and analgesia induced by acute morphine administration. Chronic morphine treatment results in development of tolerance to morphine analgesic action. The obtained data show that chronic co-administration of L-NAME prevents the development of morphine tolerance. Similar results have been presented recently.[31,32] Naloxone precipitated the typical withdrawal syndrome in morphine dependent rats. However, the characteristics and magnitude of withdrawal syndrome are very much alleviated by chronic co-administration of L-NAME. Our data add further support to the dominating viewpoint that inhibition of NOS/NO pathways could alleviate the development and severity of morphine abstinence syndrome.[33] In this study, for the first time the symptoms of morphine tolerance, dependence and withdrawal were quantified based on a novel score-point scale. This methodology helps to avoid the ambiguity of descriptive evaluation of rat behaviour.

### Cardiovascular system

The first morphine administration evoked transitory hypotension and bradycardia in all rats from group D1. The analysis showed that morphine significantly lowered SBP and DBP and decreased MBP and HR. The second morphine administration made 48 h later resulted in development of morphine tolerance manifested in negligible hemodynamic effects. The development of morphine tolerance was completely prevented in all rats pretreated with L-NAME (D2). NOS inhibition per se was manifested in moderate hypertension and bradycardia. The analysis revealed that the hemodynamic effects of the second morphine administration in the rats from group D2 matched the effects of the second morphine administration in the rats from group D1. The development of tolerance to morphine cardiovascular action in the absence and presence of L-NAME is summarized in Table 3.

**Table 3.** Changes (%) in systolic blood pressure (SBP), diastolic blood pressure (DBP), mean blood pressure (MBP) and heart rate (HR) due to first (I) and second (II) morphine application in the absence (D1) and presence (D2) of L-NAME.

	I (D1)	II (D1)	I (D2)	I (D2)
SBP	−39 ± 9	2 ± 1^a^	−46 ± 7	−39 ± 10
DBP	−59 ± 10	1 ± 2^a^	−58 ± 10	−52 ± 12
MBP	−52 ± 10	1 ± 2^a^	−52 ± 6	−46 ± 12
HR	−78 ± 6	−13 ± 6^a^	−89 ± 1	−75 ± 8

^a^ *P* ≤ 0.001 vs. I (G1).

The application of L-arginine did not lead to a significant change either in the effects of morphine in the group D3 rats, or in the effects of L-NAME on the development of tolerance to morphine cardiovascular action in the rats from group D4. The development of tolerance to morphine cardiovascular action in the absence and presence of L-NAME following pretreatment with L-arginine is shown in Table 4.

**Table 4.** Changes (%) in systolic blood pressure (SBP), diastolic blood pressure (DBP), mean blood pressure (MBP) and heart rate (HR) due to first (I) and second (II) morphine application in the absence (D3) and presence (D4) of L-NAME and pretreatment with L-arginine.

	I (D3)	II (D3)	I (D4)	I (D4)
SBP	−42 ± 9	7 ± 2^a^	−42 ± 4	−21 ± 6^b^
DBP	−58 ± 8	6 ± 4^a^	−53 ± 7	−29 ± 9^b^
MBP	−53 ± 7	7 ± 3^a^	−50 ± 10	−26 ± 6^b^
HR	−81 ± 6	−26 ± 10^a^	−71 ± 9	−62 ± 10^b^

^a^ *P* ≤ 0.001 vs. I (G3); ^b^ *P* ≤ 0.05 vs. I (G4).

These results demonstrate rapid development of tolerance to cardiovascular action of repeated morphine administration and tolerance reversal by pretreatment with L-NAME. It is likely that inhibition of the NOS/NO cascade contributes significantly for tolerance alleviation. The study, however, does not discriminate which NOS isoform inhibition is responsible for the alleviation of tolerance to the cardiovascular action of morphine. The principal cardiovascular effects of opioid peptides are peripheral vasodilation and reduced resistance, baroreceptor inhibition and bradycardia due to NOS/NO-dependent histamine release and vagal activation at peripheral and central level. Other data show that cross talk of opioids of myocardial origin and β-adrenoceptors plays an important role in local regulation in the heart [34] and recent data show that the heart produces prodynorphin and proenkephalin.[35]

### Concanavalin A-induced lymphocyte proliferation

The obtained results (Table 5) showed that chronic morphine treatment powerfully suppressed the proliferation of lymphocytes (G1 vs. G4). In contrast, the chronic L-NAME treatment significantly stimulated lymphocyte proliferation (G2 vs. G4). However, the combined chronic treatment of morphine and L-NAME did not change significantly lymphocyte proliferation (G3 vs. G4). Our results that chronic morphine treatment can inhibit lymphocyte proliferation support the well-known experimental and clinical evidence that opioids powerfully suppress the immune reactions.[36] The results showed that lymphocyte proliferation was unchanged after chronic morphine plus L-NAME treatment. It might be suggested that concomitant immune effects of morphine suppression and L-NAME stimulation coincide and neutralize each other through different pathways.

**Table 5.** Effect of chronic morphine and L-NAME treatment on lymphocyte proliferation.

Concanavalin A	Group G1	Group G2	Group G3	Group G4
125 μg/ml	2.74 ± 0.29^a^	117.62 ± 14.63^a^	28.84 ± 3.58	38.75 ± 3.77
500 μg/ml	2.26 ±1.21^a^	107.52 ± 9.70^a^	19.11 ± 2.14	23.75 ± 1.21

^a^ *P* ≤ 0.001 vs. G4.

### Thymocyte apoptosis/DNA fragmentation

**Figure 1.**
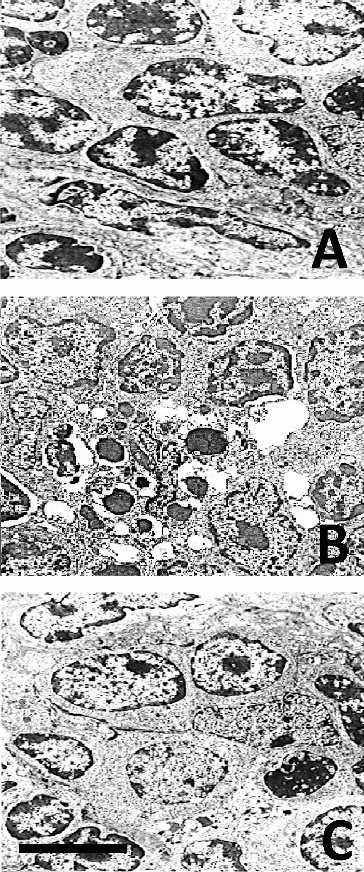
Ultrastructural data for apoptosis of thymocytes. Control (**A**); chronic morphine treatment (**B**); chronic morphine plus L-NAME treatment (**C**). Bar = 5 μm.

**Figure 2.**
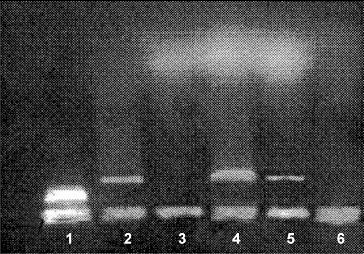
Agarose gel electrophoresis of DNA isolated from rat thymocytes. Lane 1 – DNA; lanes 2 and 4 – Control; lane 3 – chronic morphine treatment; lane 5 – chronic treatment with morphine plus L-NAME; lane 6 – DNA degrading products.

LM revealed that after chronic morphine treatment (G1) large areas in the thymus cortex contained numerous cells at different stages of apoptosis. EM showed significant cell shrinkage, chromatin rearrangement, chromatin condensation/aggregation, cytoplasmic vacuolization, disruption of the nuclear membrane and nuclear fragmentation (Figure 1(B)) as compared to the control (Figure 1(A)). The ultrastuctural findings showed chromatin marginalization (initial apoptosis) or residual bodies (advanced apoptosis). The results indicated that thymocyte apoptosis was alleviated significantly after chronic morphine plus L-NAME treatment (G3). The number of apoptotic cells in the thymus cortex was lower and less chromatin damage and nuclear fragmentation were observed (Figure 1(C)). The analysis of the obtained gel electrophoresis data revealed that after chronic morphine treatment much of the genomic DNA was fragmented and was of low molecular weight. However, after chronic treatment with morphine plus L-NAME, there was less low molecular weight genomic DNA and more unfragmented genomic DNA of higher molecular weight (Figure 2). These results agree with other data about morphine-induced apoptosis [37] mediated by the cleavage activity of caspase-3 and caspase-8.[38] Our results showed that chronic morphine plus L-NAME treatment resulted in few apoptotic cells, less low molecular weight genomic DNA and preservation of high molecular weight unfragmented genomic DNA. These data support the viewpoint that NOS inhibition could counteract apoptosis by activation of NF-κB binding.[39]

### Brain NADPH diaphorase activity

NADPH diaphorase positive cells were observed in all of the investigated brain nuclei, with the population of positive cells being most dense in PAG. The topographic distribution and morphologic patterns of these cells resembled neuronal distribution and patterns that are characteristic for other brain nuclei. The dense blue stained reaction product was observed to be localized in nerve cell bodies and processes. More PAG neurons were NADPH diaphorase positive cells with elliptical or multipolar shape and small to medium size. Less DRN neurons were NADPH diaphorase positive cells with elongated to fusiform shape and small to medium size. Many neurons in LTD and PPN were NADPH diaphorase positive cells, multipolar, triangular or elongated in shape and medium to large in size. Densitometric evaluation revealed well expressed basal level of NADPH diaphorase activity. The alterations in NADPH diaphorase activity due to *in vivo* drug treatment are shown in Table 6.

**Table 6.** Densitometric evaluation of NADPH diaphorase positive staining in brain stem nuclei.

Group	Periaqueductal grеy matter	Dorsal raphe nucleus	Pedunculopontine tegmental nucleus	Laterodorsal tegmental nucleus
G1	165.6 ± 6.1^a^ (*N* = 2192)	175.4 ± 3.4^a^ (*N* = 797)	138.4 ± 3.2^a^ (*N* = 1694)	142.7 ± 4.1^a^ (*N* = 1019)
G2	120.0 ± 4.7^a^ (*N* = 1894)	143.5 ± 7.3^a^ (*N* = 907)	117.6 ± 4.1^a^ (*N* = 1290)	121.4 ± 3.9^a^ (*N* = 598)
G3	147.2 ± 4.6^a^ (*N* = 2321)	153.6 ± 7.7^a^ (*N* = 692)	128.1 ± 4.6^a^ (*N* = 1534)	127.3 ± 5.1^a^ (*N* = 656)
G4	214.7 ± 5.5 (*N* = 1708)	226.9 ± 5.5 (*N* = 859)	208.3 ± 5.7 (*N* = 1376)	213.7 ± 6.2 (*N* = 812)
G5	241.1 ± 5.8^a^ (*N* = 2259)	241.6 ± 7.2^a^ (*N* = 807)	239.5 ± 6.7^a^ (*N* = 1902)	253.3 ± 1.1^a^ (*N* = 207)
G6	238.4 ± 5.6^a,b^ (*N* = 2458)	237.6 ± 5.9^a,b^ (*N* = 758)	237.9 ± 5.1^a,b^ (*N* = 1718)	242.5 ± 8.4^a,b^ (*N* = 900)
G7	189.5 ± 12.8 (*N* = 2043)	193.3 ± 13.4 (*N* = 930)	189.4 ± 16.7 (*N* = 1490)	201.1 ± 17.9 (*N* = 991)
G8	215.8 ± 6.9 (*N* = 2025)	217.2 ± 5.4 (*N* = 420)	219.7 ± 9.3 (*N* = 1652)	210.4 ± 15.7 (*N* = 1186)

^a^ *P* ≤ 0.001 vs. G4; ^b^ *P* ≤ 0.001 vs. G2; (N) number of revealed NADPH positive neurons.

The results show that chronic morphine treatment decreased NADPH diaphorase activity (G1 vs. G4) and equal enzyme inhibition was found after chronic morphine plus L-NAME treatment (G3 vs. G4). The inhibition of NADPH diaphorase activity was maximal after chronic L-NAME treatment (G2 vs. G4). Acute morphine administration changed insignificantly NADPH diaphorase activity and showed a trend to increase the number of NADPH diaphorase positive cells (G7 vs. G4). The results indicated that NADPH diaphorase activity was increased after naloxone-precipitated withdrawal (G5 vs. G4). The enzyme activity was similarly increased when naloxone-precipitated withdrawal was preceded by chronic L-NAME treatment (G6 vs. G4). In contrast, NADPH diaphorase activity remained unchanged after single naloxone administration in naive rats (G8 vs. G4). The results indicated that chronic morphine treatment can inhibit NADPH diaphorase. This observation is in harmony with other reports that inhibition of neuronal NOS attenuates morphine tolerance reducing p38MAPK activation.[40] An unexpected finding was that chronic treatment with morphine plus L-NAME resulted in similar enzyme inhibition. The results for equal increase of NADPH diaphorase positive staining after naloxone-precipitated withdrawal with or without preceding chronic L-NAME treatment is in agreement with other reports.[14] NADPH diaphorase activity was found to remain unchanged after single naloxone administration in the naive rats.

## Conclusions

The present research showed that chronic co-administration of NOS inhibitor L-NAME was able to retard the development of opioid tolerance/dependence, to attenuate significantly naloxone-precipitated withdrawal syndrome and to prevent the tolerance to the cardiovascular action of morphine. In agreement with this finding, the histochemical data demonstrated that chronic morphine treatment resulted in a ubiquitous inhibition of NADPH diaphorase activity in PAG, DRN, PPN and LDT, which played a pivotal role in pain processing. The results showed increased NADPH diaphorase activity in these brain nuclei after naloxone-precipitated withdrawal. Chronic morphine treatment powerfully suppressed the proliferation of lymphocytes but lymphocyte proliferation was unchanged after chronic treatment with morphine plus L-NAME. It might be suggested that concomitant immune effects of morphine suppression and L-NAME stimulation coincide and neutralize each other through different pathways. Thymocyte apoptosis was shown to be very much restricted by supplementation of chronic morphine treatment with L-NAME, as revealed by the evidence of few apoptotic cells, less low molecular weight genomic DNA and preservation of high molecular weight non-fragmented genomic DNA. In addition, the symptoms of morphine tolerance, dependence and withdrawal were quantified for the first time by a novel score-point scale. This methodology proved very useful in avoiding the ambiguity of descriptive evaluation of the behavioural symptoms.

## References

[cit0001] Williams JT, MacDonald JC, Manzoni O (2001). Cellular and synaptic adaptations mediating opioid dependence. Physiol Rev.

[cit0002] Manassi CR, Leite-Paniss CR, Menescalde-Oliveira L. (1999). Ventrolateral periaqueductal gray matter and the control of tonic immobility. Brain Res Bull.

[cit0003] Heinzen EL, Pollack GM (2004). The development of morphine antinociceptive tolerance in nitric oxide synthase-deficient mice. Biochem Pharmacol.

[cit0004] Bhargava HN, Thorat SN (1996). Evidence for a role of nitric oxide of the central nervous system in morphine abstinence syndrome. Pharmacology.

[cit0005] Bhargava HN, Bian JT (1997). N(G)-Nitro-L-Arginine reverses L-Arginine induced changes in morphine antinociception and distribution of morphine in brain regions and spinal cord of the mouse. Brain Res.

[cit0006] Bhargava HN, Bian JT (1998). Effects of acute administration of L-Arginine on morphine antinocipeption and morphine distribution in central and peripheral tissue of mice. Pharmacol Biochem Behav.

[cit0007] Pataki I, Telegdy G (1998). Further evidence that nitric oxide modifies acute and chronic morphine actions in mice. Eur J Pharmacol.

[cit0008] Dambisya YM, Lee T-L (1996). Role of nitric oxide in the induction and expression of morphine tolerance and dependence on mice. Br J Pharmacol.

[cit0009] Babey A-M, Kolesnikov Y, Cheng J, Inturrisi CE, Trifilletti RR, Pasternak GW (1994). Nitric oxide and opioid tolerance. Neuropharmacology.

[cit0010] Leza JC, Lisasoian I, Cuellar B, Moro MA, Lorenzo P (1996). Correlation between brain nitric oxide synthase activity and opioid withdrawal.. Nahunyn-Schmiedeberg's Arch Pharmacol.

[cit0011] Herman BH, Vocci F, Bridge P (1995). The effects of NMDA receptor antagonists and nitric oxide synthase inhibitors on opioid tolerance and withdrawal. Medication development issues for opiate addiction. Neuropsychopharmacology.

[cit0012] Ashina M, Lassen LH, Bendtsen L, Jensen RA, Olesen J (2000). Inhibition of nitric oxide gas synthase and analgesic effect in chronic pain. Vgeskr Leager.

[cit0013] Guney HZ, Gorgun CZ, Tunctan B, Uludag O, Hodoglugil U, Abacioglu N, Zengil H (1998). Circadian-rhythm-dependent effects of L-N^G^-Nitroarginine methyl ester (L-NAME) on morphine-induced analgesia. Chronobiol Int.

[cit0014] Kumar S, Bhargava HN (1997). Time course of the changes of central nitric oxide synthase following chronic treatment with morphine in the mouse: reversal by naltrexone. Gen Pharmacol.

[cit0015] Milichar JK, Daglish MRC, Nutt DJ (2001). Addiction and withdrawal–current views. Curr Opin Pharmacol.

[cit0016] Bhargava HN, Kumar S, Barjavel MJ (1998). Kinetic properties of nitric oxide synthase in cerebral cortex and cerebellum of morphine tolerant mice. Pharmacology.

[cit0017] Yaksh TL, Wallace MS, Brunton LL, Chabner BA, Knollmann BC (2011). Opioids, analgesia, and pain management. Goodman & Gilman's the pharmacological basis of therapeutics.

[cit0018] Pugsley MK (2002). The diverse molecular mechanisms responsible for the actions of opioids on the cardiovascular system. Pharmacol Ther.

[cit0019] Page GG, Ben-Eliyahu S (1997). The immune-suppressive effect of pain. Semin Oncol Nurs.

[cit0020] Mellon RD, Bayer BM (1998). Evidence for opioid central receptors in the immunomodulatory effects of morphine: Review of potential mechanisms of action. J Neuroimmunol.

[cit0021] Welters ID, Menzebach A, Goumon Y (2000). Morphine inhibits NF-kappaB nuclear binding in human neutrophils and monocytes by a nitric oxide-dependent mechanism. Anesthesiology.

[cit0022] Kim MS, Cheong YP, So HS, Lee KM, Kim TY, Oh J, Chung YT, Son Y, Kim BR, Park R (2001). Protective effects of morphine in peroxynitrite-induced apoptosis of primary rat neonatal astrocytes: potential involvement of G protein and phosphatidylinositol 3-kinase (PI3 kinase). Biochem Pharmacol.

[cit0023] Persson AI, Thorlin T, Bull C, Eriksson PS (2003). Opioid-induced proliferation through the MAPK pathway in cultures of adult hippocampal progenitors. Mol Cell Neurosci..

[cit0024] Belcheva MM, Szucs M, Wang D, Sadee W, Coscia CJ (2001). μ-Opioid receptor-mediated ERK activation involves calmodulin-dependent epidermal growth factor receptor transactivation. J Biol Chem..

[cit0025] Tegeder I, Geisslinger G (2004). Opioids as modulators of cell death and survival–unraveling mechanisms and revealing new indications. Pharmacol Rev.

[cit0026] D`Amour FE, Smith DL (1941). A method for determining loss of pain sensation. J Pharmacol Exp Ther..

[cit0027] Vlaskovska M, Nylander I, Schramm M, Hahne S, Kasakov L, Silberring J, Terenius L (1997). Opiate modulation of dynorphine conversion in primary cultures of rat cerebral cortex. Brain Res.

[cit0028] Marinova TT (2005). Epithelial framework reorganization during human thymus involution. Gerontology.

[cit0029] Marinova Z, Savov A, Kremenski I, Vlaskovska M (2000). Molecular and cellular alterations in rat thymus after long term treatment with morphine and L-NAME. Balkan J Med Genet.

[cit0030] Scherer-Singler U, Vincent SR, Kimura H, McGeer EG (1983). Demonstration of a unique population of neurons with NADPH-diaphorase histochemistry. J Neurosci Methods.

[cit0031] Santamarta MT, Ulibarri I, Pineda J (2005). Inhibition of neuronal nitric oxide synthesis attenuates the development of morphine tolerance in rats. Synapse.

[cit0032] Ozdemir E, Bagcivan I, Durmus N, Altun A, Gursoy S (2011). The nitric oxide-cGMP signaling pathway plays a role in tolerance to the analgesic effect of morphine. Can J Physiol Pharmacol.

[cit0033] Adams ML, Kalicki JM, Meyer ER, Cicero TJ (1993). Inhibition of morphine withdrawal syndrome by a nitric oxide synthase inhibitor, N^G^-nitro-L-arginine methyl ester. Life Sci.

[cit0034] Pepe S, Van den Brink OW, Lakatta EG, Xiao RP (2004). Cross-talk of opioid peptide receptor and beta-adrenergic receptor signalling in the heart. Cardiovasc Res.

[cit0035] Headrick JP, Pepe S, Peart JN (2012). Non-analgesic effects of opioids: cardiovascular effects of opioids and their receptor systems. Curr Pharm Des.

[cit0036] Lysle DT, Coussons ME, Watts VJ, Bennett EN, Dykstra LA (1993). Morphine-induced alterations of immune status: dose dependency, compartment specificity and antagonism by naltrexone. J Pharmacol.

[cit0037] Liu L-W, Lu J, Wang XH, Fu S-K, Li Q, Lin F-Q (2013). Neuronal apoptosis in morphine addiction and its molecular mechanism. Int J Clin Exp Med.

[cit0038] Wang J, Charboneau R, Balasubramanian S, Barke RA, Loh HH, Roy S (2001). Morphine modulates lymph node-derived T lymphocyte function: role of caspase-3, -8, and nitric oxide. J Leukoc Biol.

[cit0039] Welters ID, Menzebach A, Goumon Y, Cadet P, Menges T, Hughes TK, Hempelmann G, Stefano GB (2000). Morphine inhibits NF-kappaB nuclear binding in human neutrophils by a nitric oxide-dependent mechanism. Anesthesiology.

[cit0040] Liu W, Wang C-H, Cui Y, Mo L-Q, Zhi J-L, Sun S-N, Wang Y-L, Yu H-M, Zhao C-M, Feng J-Q, Chen P-X (2006). Inhibition of neuronal nitric oxide synthase antagonizes morphine antinociceptive tolerance by decreasing activation of p38 MAPK in the spinal microglia. Neurosci Lett.

